# Stenting for subclavian steal phenomenon to restore cerebral perfusion due to acute carotid occlusion following carotid endarterectomy: a case report

**DOI:** 10.1186/s13256-024-04546-8

**Published:** 2024-05-08

**Authors:** Shin Hirota, Masataka Yoshimura, Junshi Cho, Toshihiko Hayashi, Azumi Kaneoka, Kei Ito, Juri Kiyokawa, Shinji Yamamoto

**Affiliations:** Department of Neurosurgery, Tsuchiura Kyodo General Hospital, 4-1-1 Otsuno, Tsuchiura-shi, Ibaraki, 300-0028 Japan

**Keywords:** Carotid endarterectomy, Carotid artery occlusion, Subclavian steal, Subclavian artery stenting, Case report

## Abstract

**Background:**

Perioperative symptomatic carotid artery occlusion after carotid endarterectomy is a rare complication. In this study, we present a case of symptomatic acute carotid artery occlusion that occurred after carotid endarterectomy in a patient with coexistent subclavian artery steal phenomenon, which was successfully treated with subclavian artery stenting.

**Case presentation:**

A 57-year-old East Asian female presented with stenosis in the left common carotid artery and left subclavian artery along with subclavian steal. The proximal segment of the left anterior cerebral artery was hypoplastic, and the posterior communicating arteries on both sides were well-developed. Left internal carotid artery stenosis progressed during the follow-up examination; therefore, left carotid endarterectomy was performed. On the following day, symptoms of cerebral perfusion deficiency appeared due to occlusion of the left carotid artery. The stenotic origin of the left common carotid artery and the suspected massive thrombus in the left carotid artery posed challenges to carotid revascularization. Therefore, left subclavian artery stenting for the subclavian steal phenomenon was determined to be the best option for restoring cerebral blood flow to the whole brain. Her symptoms improved after the procedure, and the postprocedural workup revealed improved cerebral blood flow.

**Conclusion:**

Subclavian artery stenting is safe and may be helpful in patients with cerebral perfusion deficiency caused by intractable acute carotid occlusion coexisting with the subclavian steal phenomenon. Revascularization of asymptomatic subclavian artery stenosis is generally not recommended. However, cerebral circulatory insufficiency as a comorbidity may be worth considering.

## Background

Perioperative symptomatic carotid artery occlusion after carotid endarterectomy (CEA) is a rare but severe complication reported in 0.5–3.6% of patients undergoing CEA [1, 2], with severe disability in 15–28% and fatality in 5.6–44% of patients [3–5]. Thrombus removal through wound re-exploration [3, 5] and carotid artery stenting (CAS) [4, 6] have been reported as revascularization methods for such cases. However, the necessity and appropriate method of bailout intervention for post-CEA carotid occlusion remain unclear.

Subclavian artery stenosis is a rare peripheral arterial disease, with an overall prevalence of 1.5%; however, the prevalence is reported to be as high as 11–40% in patients with comorbid risk factors for atherosclerosis [7]. Typically, this stenosis occurs on the proximal side of the affected subclavian artery, wherein blood flow in the ipsilateral vertebral artery is reduced and even reversed to direct cerebral blood flow to the ipsilateral arm. This redirection of blood flow is termed “subclavian artery steal,” and when additional symptoms such as vertebrobasilar insufficiency or upper limb weakness occur, this condition is referred to as “subclavian steal syndrome. This report describes a case of cerebral perfusion deficiency caused by post-CEA carotid artery occlusion. The patient was successfully treated by addressing the concomitant asymptomatic subclavian steal phenomenon instead of performing carotid revascularization. As there are no existing reports on this bailout strategy, we will describe its utility and limitations.

## Case presentation

**Fig. 1:**
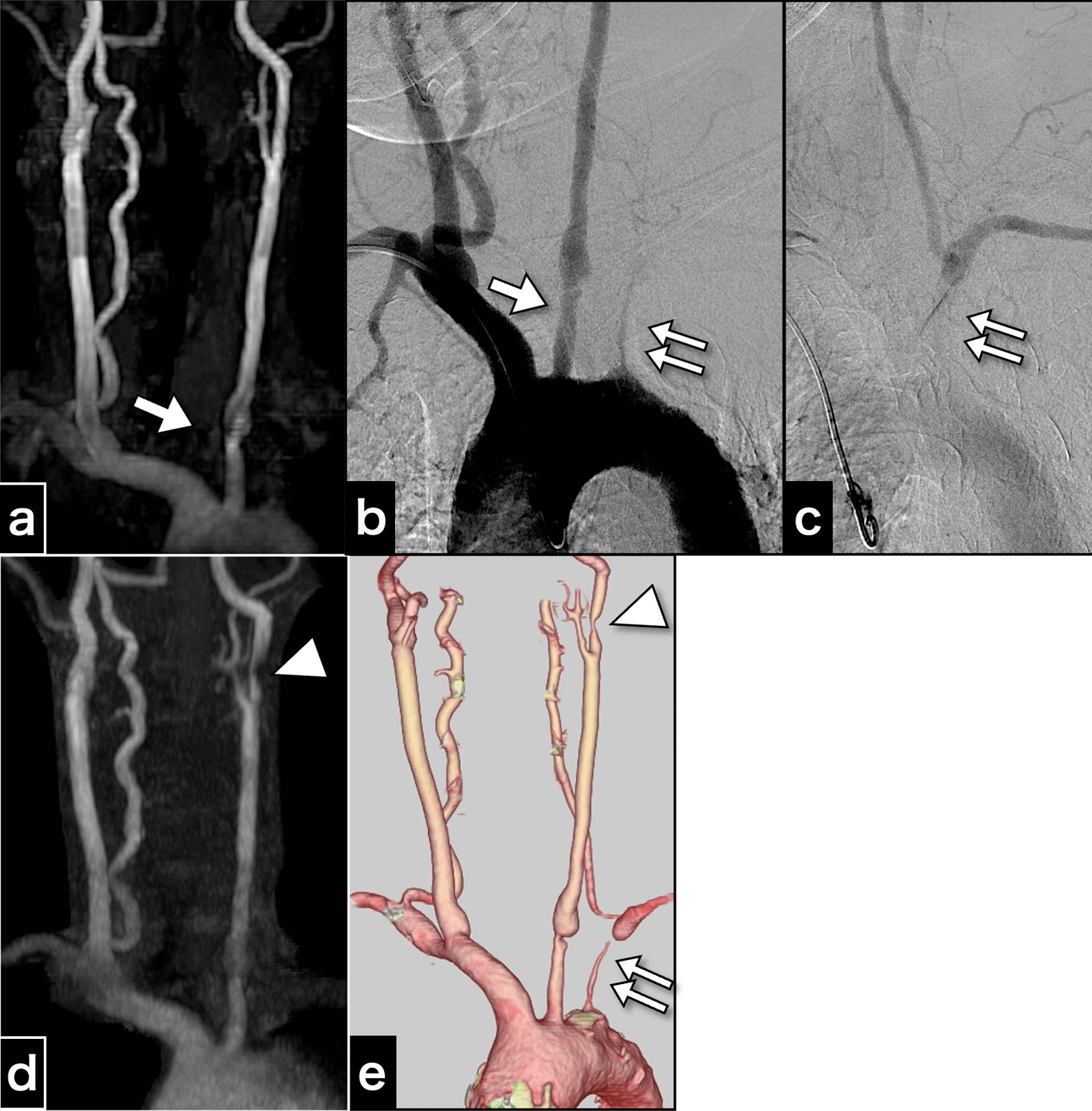
Cervical artery images obtained 3 years before carotid endarterectomy and just before carotid endarterectomy. **a** Magnetic resonance aortogram findings 3 years before carotid endarterectomy. **b** Aortogram findings 3 years before carotid endarterectomy. **c** The late-phase aortogram showing retrograde filling of the left vertebral artery and antegrade filling of the distal left subclavian artery. **d** Magnetic resonance aortogram findings just before carotid endarterectomy. **e** Computed tomography angiography findings just before carotid endarterectomy. The examinations performed 3 years before carotid endarterectomy **a–c** show an irregular origin of the left common carotid artery (arrow) and severe stenosis of the left subclavian artery (double arrow), and indications for the left subclavian steal phenomenon. The images just before carotid endarterectomy also depict progressive left internal carotid artery stenosis (arrowhead)

A 57-year-old right-handed female patient of East Asian descent, with poorly controlled atherosclerotic risk factors of dyslipidemia, diabetes mellitus, and severe hypertension, was prescribed atorvastatin calcium hydrate (5 mg once daily), and this medication regimen was maintained at the time of presentation. The patient had undergone health screening magnetic resonance angiography (MRA) (Fig. 1a). MRA revealed an asymptomatic pseudo-occlusion of the left subclavian artery and an irregular origin of the left common carotid artery (CCA). The aortogram showed left subclavian artery stenosis with subclavian steal phenomenon and irregularity in the origin of the left CCA (Fig. 1b, c). Cannulation to the CCA was avoided to minimize the risk of complications. The patient had no systemic inflammatory symptoms such as fever, arthralgia, or fatigue; vasculitis syndrome was not suspected. Because the patient was asymptomatic, no intervention was performed, and the patient was scheduled for routine follow up with medical treatment.

Three years later, MRA and computed tomography angiography (CTA) revealed new asymptomatic moderate stenosis of the left internal carotid artery (ICA) (Fig. 1d, e). Duplex ultrasonography revealed an accelerated peak systolic velocity of 217.1 cm/s. CEA was performed using a temporary intraluminal shunt.

### Post-CEA clinical course

The patient regained consciousness from general anesthesia without any neurological deficits. However, on the first postoperative day, the patient experienced intermittent left monocular vision loss. Further, she experienced somnolence, orthostatic hypotension, and vomiting resulting from the vagal reflex, triggered by the operated carotid sinus. Continuous catecholamine infusion was needed to maintain her systolic blood pressure against orthostatic hypotension and vagal reflex.

**Fig. 2:**
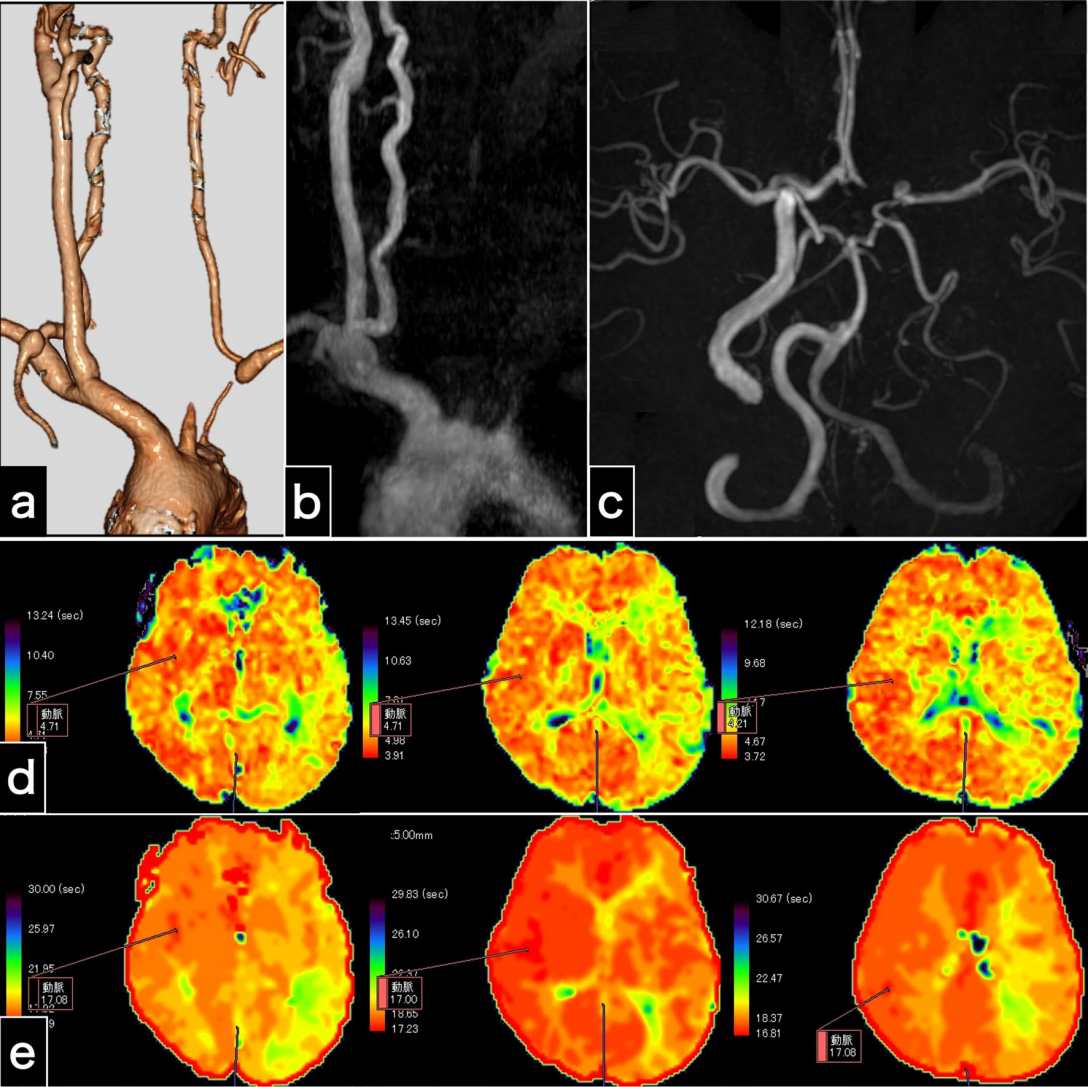
Examinations 1 day after carotid endarterectomy. **a** Computed tomography angiography image shows left carotid artery occlusion and retrograde left vertebral artery flow. **b** The cervical magnetic resonance angiography image shows left carotid artery occlusion and no left vertebral artery antegrade flow. **c** Intracranial magnetic resonance angiography indicates no intracranial vessel occlusion and substantial collateral flow through the bilateral posterior communicating arteries. **d, e** Perfusion magnetic resonance imaging findings. The maps of the mean transit time (**d**) and time to peak (**e**) illustrate prolonged times in the left cerebral hemisphere

Radiological examination (Fig. 2) demonstrated left carotid artery occlusion between the origin of the CCA and the petrous ICA, making the site and cause of obstruction unclear. Cervical CTA (Fig. 2a) and MRA (Fig. 2b, c) revealed retrograde left vertebral arterial flow, indicative of cerebral blood flow diversion (stolen) to the left arm due to the subclavian steal phenomenon. Intracranial MRA revealed an entire intradural artery with collateral flow, a hypoplastic left anterior cerebral artery (A1), and distinct bilateral posterior communicating arteries (Fig. 2c), suggesting compensatory supplementary circulation via left posterior communicating artery from the right ICA and the right vertebral artery to counterbalance the cerebral blood flow insufficiency due to left carotid artery occlusion and left vertebral artery regurgitation. Diffusion-weighted imaging revealed only two high-intensity spots. Magnetic resonance imaging (MRI) perfusion revealed symmetrical cerebral blood flow and cerebral blood volume. However, MRI perfusion revealed a prolonged mean transit time (Fig. 2d) and time to peak (Fig. 2e). These findings indicated slight cerebral blood flow insufficiency. We diagnosed the patient with left carotid occlusion and orthostatic hypotension complicated by left amaurosis fugax. Accordingly, we explored treatment options to restore cerebral circulation.

### Treatment options to restore cerebral circulation

Vascular dissection was considered as the primary mechanism of occlusion. However, we could not visualize the external carotid artery, ICA, or CCA on radiological examination (Fig. 2); therefore, the dissection site could not be identified. The tip of the inserted internal shunt, distal edge of the endarterectomy, and vascular clamping site in the CCA were considered possible injured sites. Moreover, the onset of symptoms after an overnight period indicated possible secondary thrombus formation. The thrombus at carotid bifurcation and the CCA appeared to be extensive. Primary dissection at one of the possible sites and secondary massive thrombosis caused the occlusion. Thrombectomy via re-explosion wounds may not address dissection or thrombus outside the operating field. Endovascular treatment to non-contrast-enhanced carotid arteries could exacerbate dissection or cause distal migration of massive thrombus. Moreover, there were concerns that the narrowed origin of the left CCA might cause severe complications (Fig. 1e). Although catheterization of the CCA stenosis was considered, it could potentially compromise dissection of the aortic arch and the CCA origin in patients with chronic inflammatory diseases. However, 2 weeks later, pathological diagnosis of the resected intima confirmed atherosclerosis without chronic granulomatous changes suggestive of conditions such as Takayasu arteritis and giant cell arteritis.

Because the left vertebral artery was clearly visualized on cervical CTA (Fig. 2a) and the circle of Willis was well-developed (Fig. 2c), alleviating the subclavian steal phenomenon would restore whole-brain circulation (Fig. 1b, c). We determined that typical subclavian artery stenting, known to have a high procedural success rate [7, 8], is safer than direct carotid endovascular intervention, which is not well-visualized and has multiple underlying pathologies. This finding prompted us to perform left subclavian artery stenting as a safe palliative procedure. However, if the treatment had been ineffective, we would have performed left carotid intervention.

### Endovascular treatment

On the first postoperative day, endovascular treatment was performed under local anesthesia, with 200 mg aspirin and 300 mg clopidogrel as the loading doses. A 4-Fr 17-cm sheath was placed in the left radial artery, and a 4-Fr catheter was directed to the left subclavian artery to verify the condition of the left subclavian artery, followed by systemic heparinization. Initial angiography revealed a subclavian artery diameter of 7 mm. We planned to deploy a balloon-expandable stent, a 135-cm-shaft Express LD stent (70 × 37 mm; Boston Scientific, Marlborough, MA, USA) with a 6-Fr 90-cm guiding sheath via the right femoral artery.

**Fig. 3:**
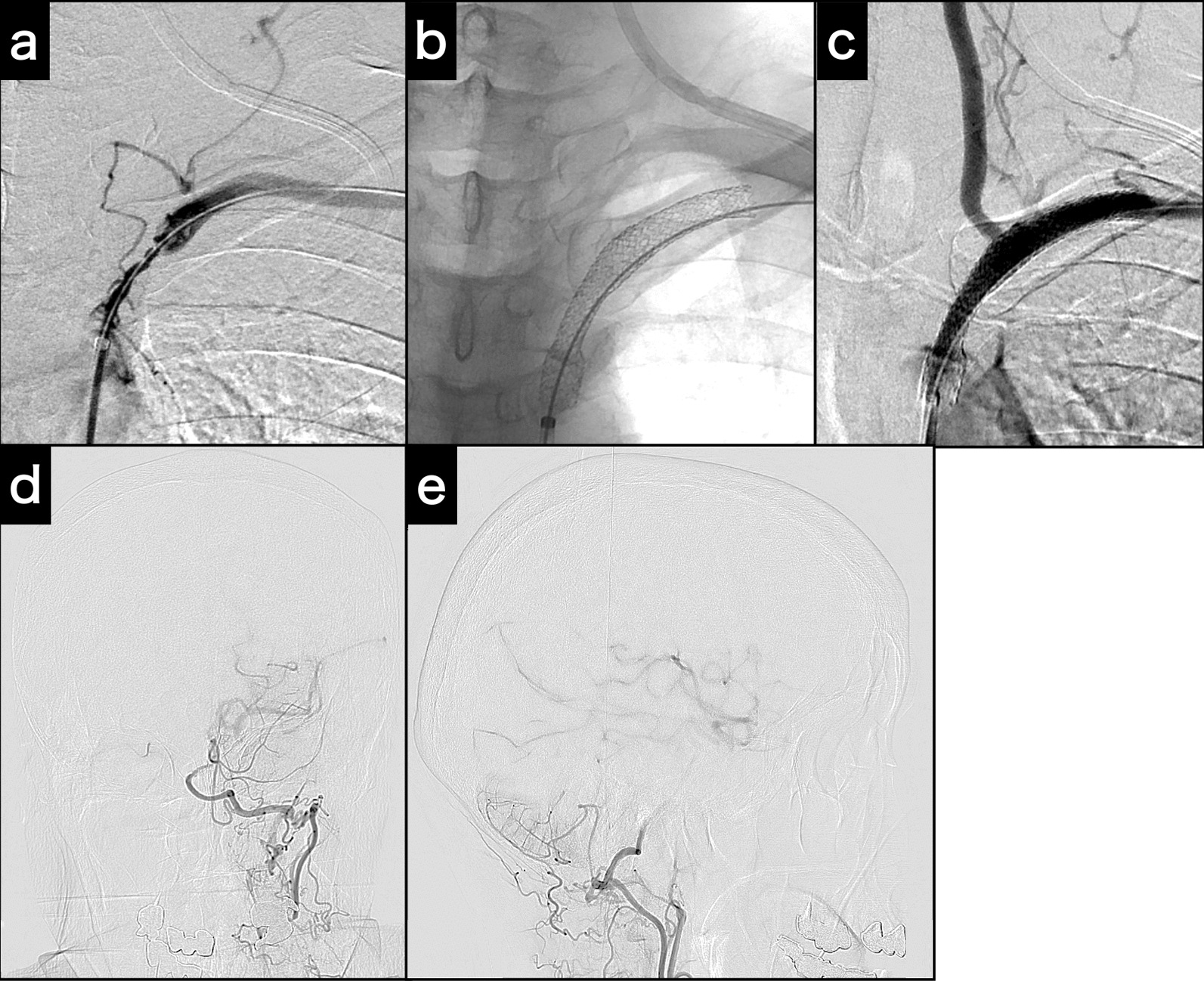
Intraoperative examinations for subclavian stenting. **a** Angiogram acquired at the completion of the pull-through technique. **b** Fluoroscopy of the deployed and expanded stents. **c** Post-stenting angiogram demonstrates potent antegrade left vertebral artery flow. **d, e** Post-stenting left subclavian artery angiogram (**d**: Town’s view; **e**: lateral view) demonstrates left internal carotid artery and middle cerebral artery via posterior communicating artery collateral flow

First, the catheter tip of a 6-Fr, 125-cm inner catheter in the guiding sheath was applied to the subclavian artery ostium. A 0.035-in., 400-cm guidewire was introduced across the stenotic lesion. Then, the guidewire was inserted into the tip of the 4-Fr catheter from the 6-Fr catheter. Finally, the 4-Fr catheter was removed from the radial sheath, and the guidewire was passed through the sheath. Thus, the pull-through technique [8] was completed from the right femoral 6-Fr guiding sheath to the left radial 4-Fr sheath (Fig. 3a). A Mustang PTA balloon catheter (3 × 40 mm; Boston Scientific) was introduced through the guiding sheath and inflated. Because of a distal vascular dissection, two Express LD stents were then applied to the subclavian artery to cover the stenosis and dissection (Fig. 3b). At the end of the procedure, left subclavian artery angiogram revealed restoration of antegrade blood flow in the vertebral artery (Fig. 3c) and left middle cerebral artery blood flow via the left posterior communicating artery (Fig. 3d, e). The angiogram showed compensation for the reduced left ICA blood flow from the left subclavian artery.

### Post-stenting clinical course

**Fig. 4:**
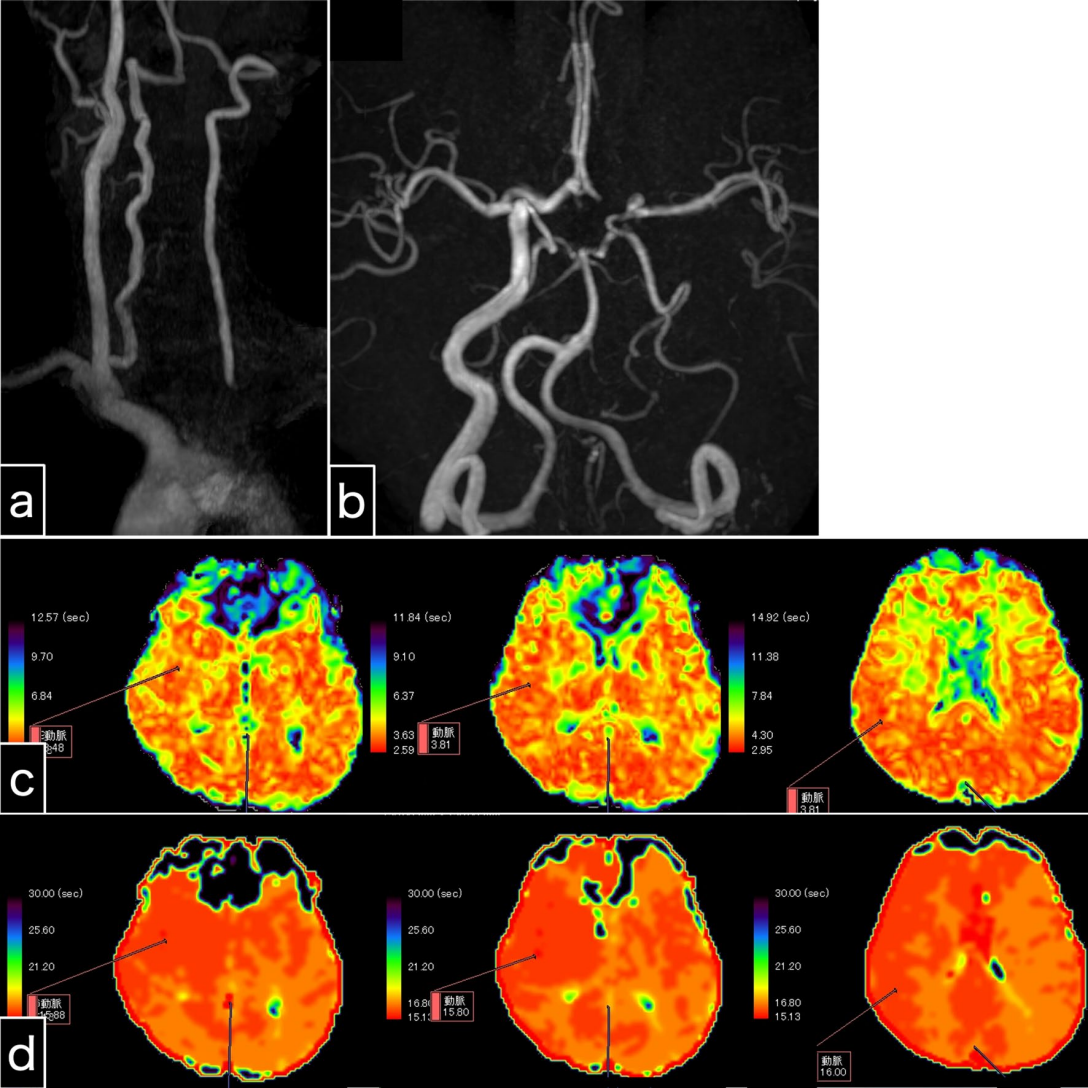
Examination 1 day after left subclavian artery stenting. **a** Cervical magnetic resonance angiography shows potent antegrade left vertebral artery flow. **b** Intracranial magnetic resonance angiography shows that the left vertebral artery intensity is stronger than that of the pre-stenting magnetic resonance angiography. The collateral blood flow via the left posterior communicating artery, and not the anterior communicating artery, is depicted. **c, d** Perfusion magnetic resonance imaging. Maps of the mean transit time (**c**) and time to peak (**d**) reveal slightly prolonged times in the left cerebral hemisphere. Perfusion magnetic resonance imaging seems to show reduced laterality

**Fig. 5:**
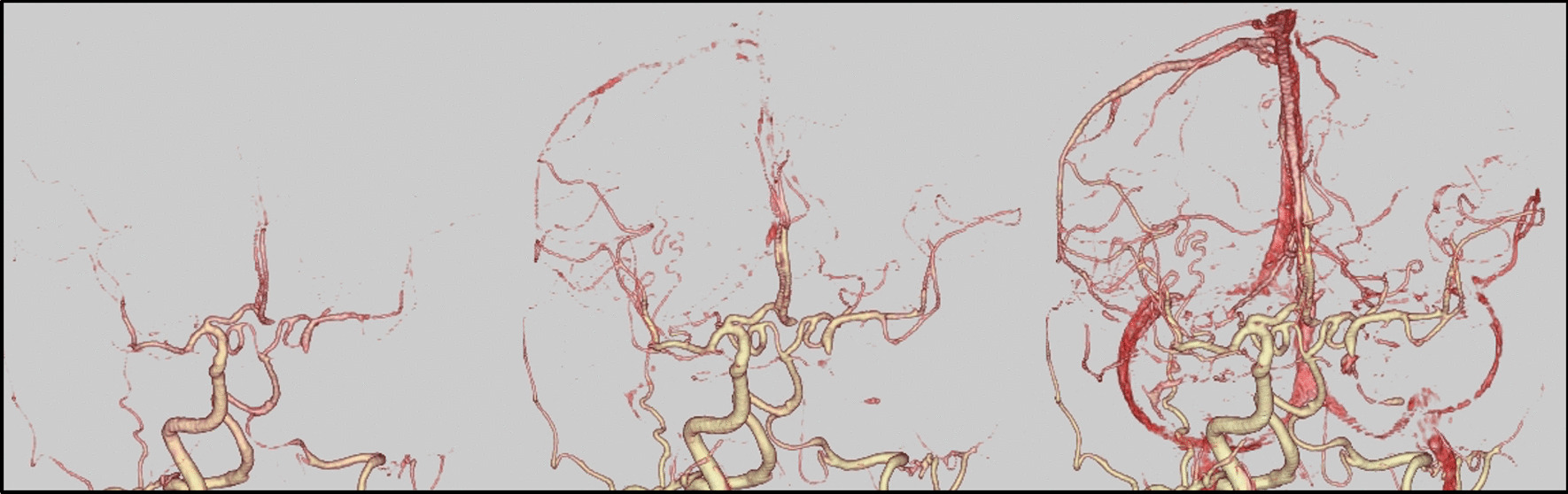
Four-dimensional computed tomography angiogram after left subclavian artery stenting. Collateral circulation is visible from the left posterior communicating artery to the left cerebral hemisphere. The left anterior cerebral artery (A1) is not visible

The amaurosis fugax resolved immediately after the stenting procedure. Somnolence and nausea resolved one day following endovascular treatment. The symptoms disappeared immediately after the present intervention, indicating their likely association with cerebral ischemia. However, orthostatic hypotension persisted, and continuous catecholamine administration was terminated 3 days following endovascular treatment. As a direct result of left subclavian artery stenting, left antegrade vertebral artery flow was clearly visible on MRA (Fig. 4a, b). As an indirect consequence, laterality appeared to be reduced on perfusion MRI when comparing the previous MRI (Fig. 2c, d) to the post-procedure MRI (Fig. 4c, d). Intracranial MRA (Fig. 4b) and four-dimensional CTA (Fig. 5) revealed collateral circulation from the left posterior communicating artery to the left cerebral hemisphere, with no collateral circulation via the left anterior communicating artery (A1). No new cerebral infarctions were observed on diffusion-weighted imaging.

The patient was discharged 2 weeks later without in-stent thrombosis. Dual antiplatelet therapy was continued for 3 months, after which aspirin alone was prescribed. For the past eight years, the patient has been attending our hospital for regular check-ups and has received prescriptions for atherosclerotic risk factors without any stroke episodes or any deficits. The patient was scheduled for routine follow-up with CTA and MRA every three months for the first year and every year thereafter. Although only a 30% width and shortest length in-stent re-stenosis have been observed, antegrade blood flow in the left vertebral artery remains maintained.

## Discussion

We described a case of post-CEA carotid occlusion successfully treated with subclavian artery stenting. Using collateral circulation, we treated the cerebral perfusion deficiency instead of direct carotid revascularization. Endovascular treatment alleviated neurological symptoms in our patient, and the patient maintained good condition over a long period. To our knowledge, this is the first report of a bail-out procedure using subclavian steal.

Severe proximal subclavian artery stenosis causes cerebral blood flow backflow from the ipsilateral vertebral artery into the ipsilateral upper arm, termed subclavian steal syndrome if this results in vertebrobasilar insufficiency or ipsilateral weakness. No surgical indications exist for asymptomatic subclavian artery stenosis in cases of subclavian steal phenomenon [9]. However, previous studies have suggested that comorbid carotid artery stenosis can precipitate the development of subclavian artery stenosis [10, 11]. Another study has also reported that 11 of 15 patients with symptomatic subclavian steal syndrome had concomitant carotid artery stenosis and that the neurological symptoms in three patients improved with CEA [11]. These findings suggest a close relationship between both vascular diseases, as they impact cerebral collateral blood flow. Moreover, both are atherosclerotic diseases, and it has been reported that one-third of patients with subclavian artery disease also have carotid artery disease [12]. The post-CEA episode in our patient further underscores the hemodynamic and pathological interconnectedness between these conditions.

We performed subclavian artery stenting to address carotid artery occlusion following thrombus formation post-CEA. We opted against direct treatment of the carotid artery due to concerns regarding an unidentified dissection site, massive thrombus, and CCA stenosis. In carotid catheter intervention, these are considered risk factors for thoracic and cervical artery dissection, sutured vessel rupture, and distal embolism. While thrombectomy with re-exploration [3, 5] and CAS [4, 6] have been reported as treatments for post-CEA carotid artery occlusion, the complications of intracranial lesions determine the prognosis for both treatment strategies. Pappada reported seven cases treated with reopening; of these, with good prognosis noted in only three cases without intracranial lesions [3]. Spiotta reported good recanalization in nine patients treated with endovascular therapy and good prognosis in three of six patients without intracranial lesions [6]. Endovascular treatment in 107 symptomatic extracranial ICA occlusive patients reported good outcomes in only 65%; moreover, distal emboli occurred in 22% [13, 14]. Although recent advancements in endovascular treatment show promise in improved outcomes for intracranial thrombectomies, distal embolization is still reported to be a poor prognostic factor [15]. Thus, preventing distal embolization was essential for achieving favorable recovery.

There was no risk of subclavian stenting causing distal dispersal of a carotid artery thrombus. The advantage of subclavian artery stenting is its high technical success rate of 95–100% and low periprocedural stroke rate of 0.8% [7, 16]. However, the restenosis rate of subclavian artery stenting is reported as 12–18.7%, which is slightly higher than that of CAS [16–20], and the indication for carotid artery revascularization is generally clear [9]. The priority of surgical intervention should be considered on a case-by-case basis. Subclavian artery stenting may be a treatment option for patients with post-CEA carotid artery occlusion, as observed in the present case. We believe that subclavian stenting was appropriate as palliative therapy.

As mentioned above, distal emboli are difficult to prevent with endovascular treatment of stenotic lesions in the proximal CCA. In this case, a combined therapeutic approach has been proposed for long lesions from the proximal CCA. In such an approach, CEA and stenting are performed from the same site toward the proximal region while preventing distal embolization by exposing the cervical carotid artery and clamping branches [21, 22]. This approach takes advantage of the benefits of CEA, which prevents distal embolization by clamping cervical vessels while using radiologic imaging to treat long lesions outside of the exposed wound site. However, the disadvantage is that the complex procedure can have a longer clamping time. If a hybrid operating room was available, the condition of our patient could have been treated safely and more radically by reopening the wound after CEA and performing angiography and endovascular treatment toward the proximal and distal sides. Using both CEA surgical instruments and endovascular devices while minimizing clamping time and maintaining a sterile environment requires advanced hybrid operating rooms and careful simulations.

Finally, palliative subclavian stenting may result in inadequate cerebral blood flow. By contrast, even without stenting, it is possible that the patient’s symptoms may have improved with medical treatment. However, the recanalization rate of intravenous thrombolysis in the extracranial ICA was low at 14.6% [23], and thrombolysis was probably inappropriate after CEA. While induced hypertension of 170–200 mmHg may be effective [24], this patient was rather hypotensive, and maintenance of marked hypertension, as reported, would have been complex. Approximately half of symptomatic extracranial ICA occlusive diseases are reported to lead to moderate or severe stroke [23], suggesting that our patient would likely have had a poor outcome without endovascular revascularization. The outcomes of these possibilities remain difficult to determine because quantitative cerebral blood flow assessments, i.e., positron emission tomography, were unavailable in the emergent situation. Moreover, sedation could be required for adequate evaluation due to impaired consciousness in some emergent cases, including the case of our patient. Sedation can induce decreasing cerebral blood flow or hypotension; therefore, it is dangerous. Performing subclavian stenting without such quantitative testing would be appropriate.

## Conclusions

We reported a case of symptomatic post-CEA carotid artery occlusion with coexistent subclavian steal. In this case, stenosis was present at the CCA’s origin, and the occlusion's mechanism and site were unknown. A large thrombus was also suspected; therefore, severe complications such as distal embolization were probable with carotid revascularization. Subclavian artery stenting without risk of carotid thrombus migration was determined to be safer for achieving revascularization of cerebral ischemia. Predicting the degree of flow restoration preoperatively is challenging, and the re-stenosis rate is slightly higher; however, the required cerebral blood flow assessments may be difficult or impossible to perform in an emergency case. Reopening the CEA wound and performing endovascular treatment in each direction while clamping the cervical artery is a safe radical technique but requires the availability of a hybrid operating room in an emergency. Large vascular disease is a systemic disease with various comorbidities, comorbid vascular disease, and collateral vascular patterns in individual cases. Therefore, subclavian stenting, which is considered a safe and effective bailout method, may be worth considering on a case-by-case basis depending on collateral circulation.

## Data Availability

The data underlying the findings of this study are available from the corresponding author upon reasonable request, with the approval of the participating investigators.

## Abbreviations

CCA: Common carotid artery
CEA: Carotid endarterectomy
CAS: Carotid artery stenting
CTA: Computed tomography angiography
ICA: Internal carotid artery
MRA: Magnetic resonance angiography
MRI: Magnetic resonance imaging
